# Molecular Insights for Precision Care in Cardiovascular Disease: Perspectives from the Special Issue

**DOI:** 10.3390/ijms27010493

**Published:** 2026-01-03

**Authors:** Michishige Terasaki, Sho-ichi Yamagishi

**Affiliations:** Division of Diabetes, Metabolism, and Endocrinology, Department of Medicine, Showa Medical University Graduate School of Medicine, Tokyo 142-8666, Japan

Cardiovascular disease (CVD) and its associated complications remain a leading cause of global morbidity and mortality in industrialized countries [1]. Dyslipidemia and its related chronic vascular inflammation accelerate the risk of development of atherosclerotic CVD, encompassing conditions like coronary artery disease (CAD), stroke, and peripheral artery disease [2]. The acceleration of atherosclerosis is significantly driven by diverse molecular signaling cascades as well as genetic predispositions that regulate lipid metabolism and arterial wall cell integrity [3]. Consequently, elucidating the molecular mechanisms underlying these initial processes is crucial, given their fundamental role in the development and progression of atherosclerotic CVD. This Special Issue highlights how emerging molecular insights into atherosclerosis are shifting the clinical focus toward individualized management strategies.

A total of twelve manuscripts were submitted to this Special Issue, each undergoing the stringent and high-standard peer-review process of *IJMS*. Among these, six outstanding contributions, including three original articles, two reviews, and one case report, were finally selected for publication in this Special Issue. It is our pleasure to introduce these curated works presented below:

Mănescu IB et al. (Contribution 1) represented a significant leap forward in the endeavor to identify high-risk individuals before the onset of symptomatic CVD by evaluating the clinical utility of an 8-SNP low-density lipoprotein cholesterol (LDL-C) polygenic score in the Romanian population. Whereas clinical focus has historically centered on monogenic familial hypercholesterolemia (FH) [4,5], this research highlighted that the polygenic form, which is driven by the cumulative effect of multiple common genetic variants, was more prevalent and deeply intertwined with premature CAD. The authors showed that combining these genetic scores with standard clinical data leads to a much clearer understanding of a patient’s actual risk. This is a crucial point because some people show symptoms very similar to FH, even though they do not have the classic single gene mutation. By validating this scoring system, the study proposes a major shift in the clinical approach: we should look at a person’s entire genetic architecture rather than searching for just a specific genetic variant. This research highlights that understanding a person’s total genetic profile is essential for personalized medicine, which can significantly reduce the long-term risk of atherosclerotic CVD. This polygenic score may have the potential to be integrated into clinical practice as a tool for personalized risk management.

Potyka K et al. (Contribution 2) provided key mechanistic insights into atherosclerotic CVD by analyzing the transcriptional activity of the tumor necrosis factor-α (TNF-α) gene and its receptors in patients with or without various degrees of coronary artery disease. While genetic risk determines a patient’s baseline predisposition to atherosclerotic CVD [6], the progression of CAD is also governed by active inflammatory molecules [7]. The authors revealed a marked elevation in TNF-α gene transcriptional activity accompanied by a progressive decrease in the activity of its receptor genes, which were associated with the severity of CAD. The observed changes in transcriptional activities of TNF-α and its receptors suggest a significant participation of the cytokine–receptor axis in the development and progression of CAD, thus indicating that these gene transcriptional activities potentially serve as useful molecular markers for detecting and evaluating CAD. The findings of Potyka et al. also suggest that TNF-α and its receptor system might be a novel molecular target for CAD and could support the personalized precision care for this devastating disorder.

Radosavljevic et al. (Contribution 3) demonstrated that alpinetin, a natural flavonoid with potent anti-oxidant and anti-inflammatory properties [8], exerted hepatoprotective effects in a C57BL/6 mouse model of alcohol-associated liver disease (ALD). Their study revealed that alpinetin significantly reduced pro-inflammatory cytokines and endoplasmic reticulum (ER) stress parameters while concurrently enhancing anti-oxidative stress markers. Furthermore, they showed that alpinetin reduced steatosis, hepatocyte ballooning, and inflammation in a mouse model of ALD. The present findings and previous observation showing that alpinetin improved high fat diet-induced metabolic dysfunction-associated steatotic liver disease (MASLD) [9] imply that alpinetin could hold substantial promise as a therapeutic agent for the management of ALD and MASLD, both of which are at increased risk of developing incident CVD [10].

Russo I et al. (Contribution 4) offered an extensive critical review regarding the pathophysiological basis of acute coronary syndromes (ACS) treatment, especially focusing on platelet biology and physiology, and the interplay between these fundamental aspects and clinical practice, which could help guide physicians in determining the best therapeutic options for the treatment of ACS. While dual antiplatelet therapy (DAPT) stands as the clinical gold standard [11], the authors described alternative therapeutic regimens to standard DAPT, with a particular focus on the clinical challenges of managing patients at high bleeding risk. Aligning pathophysiological mechanisms with international guidelines reinforces a crucial shift in cardiovascular medicine: moving away from “one-size-fits-all” protocols toward more nuanced, individualized, and safer treatment strategies for ACS.

Terriaca S et al. (Contribution 5) provided a detailed comparative analysis of microRNAs (miRNAs), which act as post-transcriptional regulators of gene expression, in atherosclerotic and non-atherosclerotic aortic aneurysms. Their study elucidated how specific miRNAs could modulate vascular smooth muscle cell phenotypes and parietal remodeling, highlighting shared and distinct molecular mechanisms of vascular wall degradation in atherosclerotic and non-atherosclerotic aortic aneurysms. This study offered essential breakthroughs by identifying miRNA signatures that distinguish these two pathological entities, offering a deeper understanding of aneurysm progression. Because the structural deterioration of the aortic wall can be characterized by greater clinical variability [12] than diameter-based clinical assessments alone might suggest, these molecular insights are critical for identifying patients at high risk of rupture. In summary, this work reinforced that miRNA expression profiles represent a fundamental cornerstone of molecular vascular diagnostics. By integrating these regulatory patterns into a patient’s entire genetic architecture, the authors advance the pursuit of precision care, enabling more accurate risk stratification and the development of targeted therapies for aortic aneurysms.

We (Contribution 6) presented an instructive case with probable sitosterolemia. This case highly suggests the necessity of molecular diagnosis for hypercholesterolemia. The patient initially presented with a phenotype of elevated serum LDL-C levels at 332 mg/dL and xanthomas. On the basis of the findings, she was suspected to have FH [13]. However, the significant beneficial effect of ezetimibe, but not rosuvastatin, made us consider the possibility that she might suffer from sitosterolemia. Targeted gene sequencing analysis identified a heterozygous *ABCG8* variant, and therefore she was diagnosed with probable sitosterolemia. Sitosterolemia is a rare autosomal recessive lipid disorder caused by mutations in the *ABCG5* and/or *ABCG8* genes, which lead to increased absorption of plant sterols. While FH is typically managed with statins, sitosterolemia requires a fundamentally different approach based on the use of ezetimibe and strict dietary modifications. Thus, it is crucial to distinguish sitosterolemia from heterozygous FH among hypercholesterolemic patients with xanthomas. The case report suggests that many patients with hypercholesterolemia remain misidentified as FH without genetic analysis and that genetic diagnostics may be indispensable for future personalized medicine in hypercholesterolemic patients.

In summary, these six contributions demonstrated that the future of cardiovascular health lies in integrating molecular insights into clinical practice. By shifting from empirical protocols to strategies informed by a patient’s entire genetic architecture, precision care will be established as a fundamental cornerstone of modern cardiovascular medicine. Ultimately, through genetic risk profiling or optimized pharmacotherapy, the transition toward molecular-based diagnostics ensures more effective, individualized interventions, ultimately redefining the standards for long-term cardiovascular stability.

## Data Availability

Not applicable.

## References

[1] GBD 2019 Diseases and Injuries Collaborators (2020). Global burden of 369 diseases and injuries in 204 countries and territories, 1990-2019: A systematic analysis for the Global Burden of Disease Study 2019. Lancet.

[2] Visseren F.L.J., Mach F., Smulders Y.M., Carballo D., Koskinas K.C., Bäck M., Benetos A., Biffi A., Boavida J.M., Capodanno D. (2021). 2021 ESC Guidelines on cardiovascular disease prevention in clinical practice. Eur. Heart J..

[3] Libby P., Buring J.E., Badimon L., Hansson G.K., Deanfield J., Bittencourt M.S., Tokgözoğlu L., Lewis E.F. (2019). Atherosclerosis. Nat. Rev. Dis. Primers.

[4] Cuchel M., Bruckert E., Ginsberg H.N., Raal F.J., Santos R.D., Hegele R.A., Kuivenhoven J.A., Nordestgaard B.G., Descamps O.S., Steinhagen-Thiessen E. (2014). Homozygous familial hypercholesterolaemia: New insights and guidance for clinicians to improve detection and clinical management. A position paper from the Consensus Panel on Familial Hypercholesterolaemia of the European Atherosclerosis Society. Eur. Heart J..

[5] Talmud P.J., Shah S., Whittall R., Futema M., Howard P., Cooper J.A., Harrison S.C., Li K., Drenos F., Karpe F. (2013). Use of low-density lipoprotein cholesterol gene score to distinguish patients with polygenic and monogenic familial hypercholesterolaemia: A case-control study. Lancet.

[6] Khera A.V., Emdin C.A., Drake I., Natarajan P., Bick A.G., Cook N.R., Chasman D.I., Baber U., Mehran R., Rader D.J. (2016). Genetic Risk, Adherence to a Healthy Lifestyle, and Coronary Disease. N. Engl. J. Med..

[7] Libby P. (2021). The changin landscape of atherosclerosis. Nature.

[8] Zhao G., Tong Y., Luan F., Zhu W., Zhan C., Qin T., An W., Zeng N. (2022). Alpinetin: A Review of Its Pharmacology and Pharmacokinetics. Front. Pharmacol..

[9] Zhou Y., Ding Y.-L., Zhang J.-L., Zhang P., Wang J.-Q., Li Z.-H. (2018). Alpinetin Improved High Fat Diet-Induced Non-Alcoholic Fatty Liver Disease (NAFLD) through Improving Oxidative Stress, Inflammatory Response and Lipid Metabolism. Biomed. Pharmacother..

[10] Moon J.H., Jeong S., Jang H., Koo B.K., Kim W. (2023). Metabolic dysfunction-associated steatotic liver disease increases the risk of incident cardiovascular disease: A nationwide cohort study. EClinicalMedicine.

[11] Byrne R.A., Rossello X., Coughlan J.J., Barbato E., Berry C., Chieffo A., Claeys M.J., Dan G.A., Dweck M.R., Galbraith M. (2023). 2023 ESC Guidelines for the management of acute coronary syndromes. Eur. Heart J..

[12] Isselbacher E.M., Preventza O., Hamilton Black J., Augoustides J.G., Beck A.W., Bolen M.A., Braverman A.C., Bray B.E., Brown-Zimmerman M.M., Chen E.P. (2022). 2022 ACC/AHA Guideline for the Diagnosis and Management of Aortic Disease: A Report of the American Heart Association/American College of Cardiology Joint Committee on Clinical Practice Guidelines. Circulation.

[13] Tada H., Nohara A., Inaz A., Sakuma N., Mabuchi H., Kawashiri M.A. (2018). Sitosterolemia, Hypercholesterolemia, and Coronary Artery Disease. J. Atheroscler Thromb..

